# Patrones de conducta social de trabajadores informales durante eventos extremos: lecciones de la vida social durante la pandemia de covid-19 en Lima, Perú

**DOI:** 10.18294/sc.2023.4494

**Published:** 2023-09-11

**Authors:** Juan Arroyo-Laguna, Carlos Eduardo Aramburú

**Affiliations:** 1 Autor de correspondencia: Doctor en Ciencias Sociales. Docente, Pontifica Universidad Católica del Perú. Investigador, Instituto de Analítica Social e Inteligencia Estratégica, Pontifica Universidad Católica del Perú, Lima, Perú. arroyo.juan@pucp.pe Pontificia Universidad Católica del Perú Instituto de Analítica Social e Inteligencia Estratégica Pontifica Universidad Católica del Perú Lima Peru arroyo.juan@pucp.pe; Pontifica Universidad Católica del Perú; 2 Master of Science. Master en Demography. Decano y Docente, Facultad de Ciencias Sociales, Pontificia Universidad Católica del Perú, Lima, Perú. caramburu@pucp.pe Pontificia Universidad Católica del Perú Facultad de Ciencias Sociales Pontificia Universidad Católica del Perú Lima Peru caramburu@pucp.pe

**Keywords:** Pandemias, Equilibrio entre Vida Personal y Laboral, Vulnerabilidad Social, Investigación Cualitativa, Perú, Pandemics, Work-Life Balance, Social Vulnerability, Qualitative Research, Peru

## Abstract

El estudio analiza los cambios en la vida social durante la pandemia y en la inicial pospandemia, en una población de alta informalidad en Lima, en la zona textil de Gamarra, que involucra a 89.123 personas. Es una investigación cualitativa, basada en 62 entrevistas semiestructuradas a confeccionistas, comerciantes de tienda y vendedores/as ambulantes. El estudio identifica dos momentos: a) las experiencias de recepción del shock por la pandemia de covid-19, y b) las experiencias de reprocesamiento del trabajo y la vida cotidiana y las salidas encontradas por la población. Se concluye que el futuro podría tener elementos adquiridos de la vida cotidiana con el covid-19, hacia una sociedad más preventiva ante los riesgos emergentes, en particular, conductas más higienistas y consideradas con la salud, más cercanas a la familia, con un uso mayor de la digitalización y trabajo híbrido, con más capital social.

## Abstract (en)

This study analyzes changes in social life during the pandemic and the early stages of the post-pandemic period within a population characterized by high levels of informality in Lima, particularly the textile district of Gamarra, encompassing 89,123 people. A qualitative approach was adopted, based on 62 semi-structured interviews with garment manufacturers, in-store traders, and street vendors. Two distinct moments could be identified: a) experiences confronting the shock induced by the Covid-19 pandemic; and b) experiences of reconfiguring work and daily life, alongside strategies adopted by the population. In conclusion, the study suggests that some elements of daily life acquired during the Covid-19 pandemic may carry over into the future, inducing society to place greater emphasis on preventive measures against emerging risks, including behaviors related to hygiene and health, fostering closer family ties, increased utilization of digitalization and hybrid work models, and the cultivation of enhanced social capital.

## INTRODUCCIÓN

El presente estudio analiza los cambios en la vida social en una población de trabajadores informales de Lima en un contexto de emergencia como el vivido con la pandemia del covid-19. Se estudia un caso de Perú, pero es un problema que excede sus fronteras. Para 2020, según cifras de la Organización Internacional del Trabajo (OIT), Colombia, Ecuador, Perú, Paraguay, El Salvador, Guatemala y Bolivia superaban el 60% de fuerza laboral informal, estando Brasil, Argentina, Panamá y República Dominicana por encima del 45%. Solo Uruguay, Chile y Costa Rica tenían entre 23% y 36% de población laboral informal^1^. Esta amplia franja poblacional latinoamericana informal afrontó la grave crisis de la pandemia desde esta condición de precariedad, y analizar su particular tipo de respuesta social es de alta relevancia para eventos extremos en el futuro. Según una buena parte de la literatura, hubo años de retroceso en la universalización de los servicios públicos, con programas de transferencias básicas para los sectores más pobres y debilitamiento de los Estados prestadores, lo que dejó en manos de las poblaciones muchas soluciones ante la pandemia^2^^,^^3^.

La respuesta de las sociedades al covid-19 muestra la proactividad de las poblaciones para aminorar el impacto de la crisis, a través de redes solidarias para alimentación, apoyos mutuos para el cuidado de menores y pacientes, enseñanza de formas de digitalización de actividades comerciales, modalidades de economía solidaria, entre muchas otras respuestas sociales^4^^,^^5^. Se abrió lo que Sánchez denominó un capítulo de lucha por la vida, que debió descansar en la innovación^4^. En el caso de Brasil, ante la persistente negligencia y negación de la gravedad del covid-19 por parte del gobierno de Bolsonaro, los residentes de muchas de las favelas de Brasil se vieron obligados a organizar sus propias respuestas a la pandemia, como imponer sus propios toques de queda^6^. Asimismo, los líderes comunitarios recaudaron fondos con el objetivo de distribuir alimentos, mascarillas y kits de higiene; incluso, usaban megáfonos para informar a los residentes sobre el correcto uso de equipos de bioseguridad, el distanciamiento físico y el lavado de manos^7^. Otros estudios en México y Argentina revelan que los residentes de áreas urbanas vulnerables dependían en gran medida unos de otros a través de los sistemas locales de ayuda mutua para abordar la crisis por covid-19. Por ejemplo, en ciudades como Buenos Aires se crearon los comités de crisis, cuyo papel fue asistir, realizar demandas al Estado y articular con otras instituciones^8^; mientras que, en México, las comunidades indígenas se organizaron y ayudaron mutuamente e implementaron soluciones locales ante los desafíos por la crisis del coronavirus^9^. En ese sentido, los grupos sociales vulnerables (favelas en Brasil, “villas” en Argentina, y las comunidades indígenas en México) gestaron sus propias iniciativas de respuesta a la covid-19, muchas veces en colaboración con organizaciones no gubernamentales y entre ellos.

La relevancia del estudio en Perú de estos patrones de conducta social durante eventos extremos se refuerza por el hecho de que Perú ocupa el primer lugar en el mundo en la tasa de fallecidos por millón de habitantes^10^, lo que expresa la fuerza del impacto de la pandemia; además ha tenido una de las cuarentenas y etapas de inmovilización general más largas a nivel internacional, prácticamente un año y medio desde el 15 de marzo del 2020^11^, cuyo efecto sobre la población ha sido importante; y el área del estudio, la zona textil de Gamarra, es además un espacio laboral de trabajadores sin un régimen laboral sujeto a la legislación estatal.

Perú tiene 33.396.700 habitantes aproximadamente^12^, con una tasa de informalidad del 73,9%^13^, con una percepción de desigualdad entre ricos y pobres del 72%^14^ y un índice de Gini para 2021 de 0,402, por debajo del promedio de América Latina del 0,464^15^. Lima tiene un tercio de la población de Perú, y cuenta con el sector informal más grande de comercio y fabricación textil, llamado “Emporio Comercial de Gamarra”. Este está ubicado en el distrito de La Victoria, en donde trabajan más de 80.000 personas reunidas en 39.000 empresas^16^. En general, la población peruana se caracteriza por ser mayoritariamente de escasos recursos, y tuvo que combatir la pandemia del covid-19 en esas condiciones. 

Dado que en el futuro es probable que debamos afrontar nuevos shocks de diferentes fuentes, es necesario identificar y analizar los patrones de conducta social y cambios en la sociabilidad de la gente, durante eventos extremos. Esto es aún más importante en los países de bajos y medianos ingresos -como el peruano- porque en ellos no existen Estados con regímenes de protección social universal^17^^,^^18^.

La revisión de la literatura realizada nos indicó otras posibilidades de interpretación de la conducta popular en la pandemia, diferentes, en algunos casos complementarias. Una es el énfasis sobre el desajuste o desviación ante sucesos críticos como sinónimo de un problema de salud mental^19^^,^^20^. En realidad, este enfoque, si se toma en forma excluyente, no tomaría en cuenta que la vida de la gente es una continua construcción y deconstrucción de formulaciones simbólicas, costumbres y respuestas afectivas ante los sucesivos cambios que trae la vida ^(^^21^^,^^22^. Ameritaría todo un estudio aparte evaluar esta interpretación medicalizada y patogénica de una parte de los especialistas en salud mental. Los nuevos fenómenos sociales que el mundo ha comenzado a vivir y observar no se pueden entender solo clasificándolos bajo la antinomia normal/anormal. La dinámica de la vida social actual conlleva, sin embargo, emociones y muchas veces estrés^23^^,^^24^.

Una segunda interpretación, complementaria si no se la toma unilateralmente, es la que otorga todo el peso de las soluciones ante la pandemia al Estado, subestimándose o incluso olvidándose el papel de la población. Una parte de la literatura ha venido mirando mucho hacia arriba y muy poco hacia abajo^25^^,^^26^. Sin embargo, la dinámica de la respuesta a la COVID-19 no se reduce a la actuación del Estado, sin desvalorizar su enorme mérito y necesidad y este estudio trata de recoger la experiencia de los que vivieron y resistieron la pandemia. 

Desde esa mirada se recogen los enfoques de la dinámica y cambio social de Kurt Levin^27^, de Berger y Luckman^28^, y el desarrollo del concepto de agencia propuesto por Olivier de Sardan y su antropología centrada en el actor^29^. El estudio recoge igualmente los aportes de Erving Goffman quien estudia la interacción social como una dramaturgia de roles y conductas esperadas, enfoque diferente del holismo estructural y del individualismo metodológico. Goffman construye una arquitectura microsociológica de la vida social basada en los conceptos de rutinas, roles, estatus, funciones sociales, personajes, expresiones verbales y no verbales, relaciones sociales, conductas creyentes y cínicas, fachadas, modales, apariencias, auditorios y escenificación^30^. Al igual que Goffman, asumimos el concepto de Simmel acerca de la sociedad como un movimiento constante de socialización y desocialización^31^. Introducimos la matriz goffmaniana al interior del modelo multietapas de la resiliencia social de Keck y Sakdapolrak, que organiza los cambios ante los shocks en tres momentos: recepción, procesamiento y salida^32^. Lo peculiar de la cuarentena como respuesta al covid-19 desde este enfoque es que desarmó bruscamente las rutinas previas y se abrió terreno a la autoasignación de roles y rutinas aún no construidas socialmente. Hasta ahí se había socializado una sabiduría sobre lo que se podía y lo que no se podía hacer. Pero, en ese momento, como vamos a analizar, la sociedad se introvirtió hacia las familias, fundiéndose vida laboral y hogareña. La pandemia representó así una sucesión de sucesos disruptivos, en que la interacción llegó a detenerse en un punto de confusión y desconcierto, hasta que retorna a la normalidad, pero con rasgos de dicha etapa.

Ante este escenario, el estudio avanza en identificar las continuidades y cambios en la sociabilidad de los trabajadores informales ante la pandemia y discute su sostenibilidad en la pospandemia. 

## METODOLOGÍA

El diseño metodológico responde a un estudio cualitativo cuyo objetivo fue rescatar las experiencias de vida de las personas que trabajan en el Emporio Comercial de Gamarra, ubicado en el distrito de La Victoria en Lima, durante la pandemia por covid-19. Se seleccionó a los trabajadores de la zona textil de Gamarra, debido a que es uno de los focos más representativos de la informalidad en Perú, constituido por 89.123 personas^33^. Es un núcleo duro de la informalidad en que el trabajo, las ventas y las remuneraciones no se hacen necesariamente sobre la base de contratos escritos^34^^,^^35^, por lo que presenta las condiciones de un laboratorio social para el estudio de la resiliencia social en situación de informalidad.

Para el estudio de los cambios en la vida social durante la pandemia se realizaron entrevistas semiestructuradas a tres tipos de informantes claves del Emporio Comercial de Gamarra: a) confeccionista; b) comerciante de tienda; y c) vendedor/a ambulante. La muestra y el reclutamiento fue establecido por tipos de informantes en las secciones o cuadrantes en que se divide la zona de Gamarra, de tal forma que se pudiera recoger una distribución amplia de vivencias. Se entrevistó a 62 informantes, de los cuales el 43% eran varones y el 57% mujeres, con edades entre los 18 y 65 años. Para la validación del instrumento de recolección de información se realizó un piloto de manera presencial, que se implementó a inicios del mes de marzo del 2021 y tomó una semana. La aplicación de las entrevistas contempló la firma de un consentimiento informado de parte de los entrevistados. El comité de ética de la Pontificia Universidad Católica del Perú aprobó el proyecto de investigación. Todas las entrevistas se realizaron de manera presencial durante julio y agosto de 2021.

Para el proceso de producción y análisis de datos cualitativos se transcribieron los audios de las entrevistas en archivo Word. Se renombraron los títulos de los archivos, ya que se anonimizaron y se convirtieron en archivos formato rtf. Se siguió con los pasos de tratamiento de datos propuestos por Cohen^36^, pero con soporte del programa Atlas Ti. Se realizó un análisis de contenido temático y la codificación de la información de las entrevistas siguiendo un proceso inductivo y se elaboró un libro de códigos. Los códigos fueron luego agrupados en categorías y subcategorías relacionadas a las dimensiones de análisis. Se utilizó la conceptualización de Goffman sobre la vida social y sus interacciones cotidianas, y de Keck y Sakdapolrak sobre las dimensiones de la resiliencia social (afrontamiento, adaptación y transformación)^32^.

## RESULTADOS

Para la exposición de los resultados, hemos adaptado dos momentos: a) las experiencias de recepción del shock por la pandemia de covid-19, y b) las experiencias de reprocesamiento del trabajo y la vida cotidiana y las salidas encontradas por la población.

### Las experiencias de recepción del shock por la pandemia de covid-19

#### Cadenas de shocks

¿La pandemia fue un solo shock o una cadena de shocks? ¿Fue un solo momento traumático o una sucesión de eventos de impactos diferenciados? Tomando en cuenta las percepciones de los entrevistados, la pandemia por covid-19 no fue sentida como un acto traumático, sino como un proceso traumático. Respondiendo a las preguntas, se trató de una cadena de shocks o sucesión de eventos de impactos diferenciados. La narrativa simplificada de la pandemia, como una sola fractura, es irreal. Durante dos años se vivió lo que Simmel sintetizó como un movimiento constante de socialización y desocialización^31^. 

El primer gran shock nacional fue la declaratoria de Estado de emergencia y el encierro total desde el 15 de marzo de 2020. Esta fue una experiencia inédita en la vida personal de toda la población en el mundo. Sin embargo, hubo otras experiencias en Perú: el shock del 4 de abril de 2020, cuando se prohibieron los velatorios de los familiares fallecidos por covid-19; el shock del 14 de abril del 2020, cuando comenzaron a escasear las camas en las unidades de cuidados intensivos y se advirtió que no se tendría el apoyo del sistema de salud en caso de contagiarse; el shock del 27 de abril del 2020, cuando el sector más pobre comenzó a colocar banderas blancas en los techos de sus hogares como señal de hambre y empezaron a emerger más ollas comunes; el shock del 21 de mayo, cuando se descubrió que los hospitales no tenían suficiente oxígeno y las familias tuvieron que movilizarse masivamente para conseguir balones por sus propios medios; y así algunos sucesos más. Lo particular de una sociedad informal y desigual es que la población sabe de antemano que no va a contar con un gran respaldo del Estado.

*La verdad, me chocó bastante, yo vine a Lima a trabajar y una vez que escuché al presidente decir que nadie va a salir de su casa, me quedé en shock. Me preguntaba “qué hago, estoy aquí con mi madre, pero qué hago” fueron como tres meses, era muy difícil, pues no sabía de dónde sacar dinero, no sabía qué hacer, acababa de regresarme, pero veía en la tele c*ó*mo se regresaban a pie y eran terribles las condiciones de cómo regresaban a sus pueblos.* (Confeccionista 4, varón, 24 años)

#### Sorpresa y sensación de vacaciones

La primera impresión ante la declaratoria de inmovilización general fue una sensación de sorpresa y de vacaciones obligadas, sobre todo para los trabajadores. Empero, finalizada la sensación de vacaciones se desplegó una cadena de temores propios de la pandemia por el covid-19: el gran temor y hasta pánico al contagio y a la muerte; el temor a pasar hambre, al despido laboral o desocupación absoluta, a la falta de oportunidades de ingreso diario (clave para los informales), a la no atención profesional sanitaria, al riesgo consciente del trabajo en plenas olas de contagio y, en general, a la incertidumbre. Como enseñara Goffman, la vida cotidiana es un entramado de rutinas, roles, estatus, funciones sociales, que quedaron suspendidas al inicio de la pandemia, originando cierta sensación de vacío^30^.

*Los primeros días los tomé como unas vacaciones. Los 15 días de la primera cuarentena eran como unas vacaciones un poco largas con la familia. Como habíamos trabajado casi todo el año, sin descanso, decidimos que estaba bien y como habíamos ahorrado, al principio, cocinábamos, jugábamos o veíamos películas, como unas vacaciones. Luego fueron pasando los días, otros 15 días más, y sentimos ya un poco de preocupación. De ahí, 15 días, ya me sentía un poco más preocupado y psicológicamente te estresas, ya que estás en casa. Fue impactante porque no estás acostumbrado a eso, porque antes salías a la hora que querías*. (Confeccionista 6, varón, 29 años)

#### Percepción de encierro: espacio y tiempo

Los primeros días de la cuarentena por covid-19 implicó un encierro forzoso para quienes estaban acostumbrados a trabajar fuera de casa: la gran mayoría. Esta situación extraordinaria tuvo serias implicancias en una distorsión del tiempo y el espacio, y la alteración de las emociones de las personas. Por un lado, conllevó a la desorganización de la rutina y cambió el sentido de equilibrio que esta otorga a las personas. Por ejemplo, el horario del sueño se vio alterado, se dormían más horas en la mañana y en la tarde, teniendo dificultades para conciliar el sueño en la noche. También se presentaron casos de insomnio que atribuyeron al encierro prologando.

*Dormía en el día, dormía en la noche, a veces no sabía a qué hora dormía porque prácticamente mi horario había cambiado; a veces dormía en la tarde, pero en la noche ya no*. (Vendedor ambulante 12, varón, 26 años)

*Fue un rompecabezas, una revolución total con respecto al sueño, tenía una costumbre de dormir desde las diez de la noche hasta las ocho de la mañana. Sin embargo, como estábamos en la situación de todos encerrados, por ejemplo, dormía a la una de la mañana, a las tres de la mañana, a las cuatro de la mañana y me levantaba a las once de la mañana, al mediodía o a la una de la tarde, porque me acostaba tarde viendo televisión, no teníamos que hacer*. (Vendedor ambulante 10, varón, 29 años)

#### Crisis de lo cotidiano y sentimientos encontrados

Por otro lado, luego de los primeros días y semanas, las personas entraron en una fase de sentimientos encontrados^37^, entre el dolor y la acción, el aislamiento de la persona contagiada y el afecto hacia ella, el miedo al contagio y la necesidad de quebrar la cuarentena para alimentarse. En la vida social, saber por lo menos el futuro inmediato, otorga seguridad. La lectura goffmaniana de la sociedad cotidiana es que esta es la escenificación de conductas y resultados esperados. Este elemento faltó en la primera etapa de la pandemia, hasta que se encontró la vacuna, que otorgó cierta seguridad de que el túnel tenía salida.

*El confinamiento total fue horrible, porque era como si no tuvieras el control. Las clases virtuales eran un rato, pero me la pasaba comiendo, mirando televisión, durmiendo a cualquier hora, pues como parabas en tu casa ya no tenías un horario establecido. Me engordé y me dio depresión por tanto tiempo en casa, un tiempo así y fue bien fuerte*. (Comerciante de tienda 1, mujer, 20 años)

*Hubo un tiempo donde llegó la depresión, la preocupación y el estrés, la verdad uno no puede estar tranquilo sin trabajar, sin hacer realmente lo que quieres hacer, ya sea estudiar, trabajar o emprender, lo que sea*. (Vendedor ambulante 12, varón, 26 años)

### Las experiencias de reprocesamiento del trabajo y la vida cotidiana y las salidas encontradas por la población

#### Estrategias de sobrevivencia

La cuarentena por covid-19 paralizó la actividad de los trabajadores del Emporio Comercial de Gamarra durante un periodo aproximado de cuatro meses. Como parte de la segunda fase de reactivación económica contemplada por el gobierno peruano, Gamarra abrió sus puertas progresivamente desde del 22 de junio del 2020. La pandemia había cortado abruptamente el característico *trabajolismo* peruano, muy presente en Gamarra^38^.

*Lo que más me gustaba era trabajar y estar en lo que a mí más me gusta, pero con la pandemia estaba bastante aburrida y estresada, porque ya no trabajaba, no ingresaba dinero y la preocupación iba creciendo día a día*. (Confeccionista 9, mujer, 28 años).

*La cuarentena afectó el horario del trabajo, porque antes de la cuarentena trabajamos hasta un poco más tarde, entre las 9 y 10 de la noche, o cuando había más trabajo nos quedábamos de amanecida. Sin embargo, con la cuarentena cerraban antes los centros comerciales y teníamos que irnos antes.* (Confeccionista 15, varón, 18 años)

Durante los primeros cuatro meses de la cuarentena, la situación fue crítica, los trabajadores no percibieron ingresos y casi no hubo ventas. Por ello, los trabajadores ambulantes, vendedores y confeccionistas adoptaron diversas estrategias de sostenimiento económico para solventar los gastos de manutención. La gran mayoría de ellos utilizó ahorros personales, recurrió al apoyo económico de sus familiares o solicitó préstamos bancarios o cambió de trabajo. Algunos quebraron y otros se vieron forzados a regresar a la venta ambulatoria. En una menor proporción, los confeccionistas se acogieron al retiro voluntario de aportes en el sistema privado de pensiones o vendieron los activos que poseían (máquinas de costura o artículos personales) para subsistir. Así, el concepto original de “estrategias de sobrevivencia”, que surgió a partir de los marginados y pobres -y luego fue sistematizado para América Latina por Adler^39^-, en la pandemia se amplió a casi todos, pues la amplia mayoría que no tiene ingresos asegurados sin trabajar debió virar a nuevas formas de aseguramiento del sustento, fuesen ambulantes, confeccionistas o comerciantes formales.

*El primer mes tenía un guardadito ahí, después recibí un préstamo del banco, así que me pude sostener un par de meses e hice unos servicios de unas cositas. Luego, hice unas mascarillas y al rato llegó el bono que también me salvó, el primer bono porque después no recibí más. También otras empresas me llamaron para hacer mamelucos y capas, cosas que eran de bioseguridad. Después, cuando recibí otro préstamo del banco al año, saqué mamelucos y de ahí me puse a hacer, hacer y me puse a vender, porque a veces no hay trabajo.* (Vendedor ambulante 14, varón, 49 años).

*En casa tenía dos máquinas, con las dos máquinas trabajé en casa, pero tuve amigos que hasta vendieron unas cuantas máquinas para estar sobrevivir, porque en verdad fue algo impactante*. (Confeccionista 6, varón, 29 años)

#### Recorte del consumo de productos y servicios

La emergencia por covid-19 incidió en el recorte de servicios y productos no prescindibles por parte de las y los trabajadores textiles de Gamarra. Como señala la mayoría de las personas entrevistadas, debieron recortar la adquisición de productos y servicios para subsistir, puesto que, a la par, se produjo un incremento de los precios de los alimentos y desabastecimiento. La mayoría optó por la reducción de gastos en servicios de entretenimiento y en la adquisición de ropa y alimentos.

*Tuve que gastar forzosamente casi todos mis ahorros en los tres meses que hubo confinamiento y fue muy difícil, porque yo vine a Lima por temas económicos y para mí me resultaba muy difícil. A veces me preguntaba por qué me fui, por qué estoy aquí, me arrepentí bastante. A veces pensaba en irme y estar con mis amigos juntos, aunque sea comiendo poco, pero estando juntos. Casi todo el ahorro lo gasté en comida, ya cuando se abrió todo, en el mes de junio o julio, no recuerdo bien, como que pude respirar recién, como que veía un poco de dinero, trabajando aquí en Gamarra, pero para mí era muy insuficiente no poder generar más*. (Confeccionista 7, varón, 24 años)

*Tuve que priorizar los gastos a los que estaba acostumbrado, como estudiante y joven hay muchas cosas que nos gustan a nosotros, pero con la pandemia se priorizó el pago de internet, luz, agua y casa*. (Vendedor ambulante 9, varón, 20 años)

#### Cambios laborales y/o empresariales

Con la ampliación de la cuarentena, algunos trabajadores optaron por no acatarla, debido a que sus fuentes de ahorro se agotaron rápidamente. La mayoría buscó fuentes de ingresos alternativos sin importar la exposición al contagio. Las personas trabajadoras con condiciones de vida más precarias, como aquellas que vendían en forma ambulante, retomaron la actividad laboral incluso antes de que se levantara la cuarentena. A nivel nacional, 235 mercados fueron clasificados como focos de contagio y primera prioridad para la prevención y control^40^. Gamarra tenía ese perfil. Las personas que vendían en tiendas buscaron reinventarse, incursionando así en la fabricación y venta de mascarillas por Internet o en el comercio ambulante desde sus hogares. La mayoría de las personas que confeccionaban indumentaria optó por retomar la actividad haciendo prendas de protección personal en sus casas, para su posterior comercio por *delivery*. La crisis se convirtió también en una oportunidad y la adaptación ante el cambio o capacidad para la innovación fueron claves para salir adelante.

*Al inicio, algunos vendedores me cuentan que fue feo, porque no han vendido nada y obviamente han tenido que tener bastante paciencia. Otros han tenido que cambiar de rubro, han aperturado sí, pero ya no con el mismo rubro; también conozco personas que vendían en mí mismo rubro, vestidos, que siguen perseverante pero las ventas son muy bajas.* (Vendedor ambulante 10, varón, 29 años)

*Para las personas que contaban con maquinaria en Gamarra les ha sido más difícil porque ha estado cerrado y no han podido trabajar en tiempo de cuarentena, pero las personas que han tenido en su propia casa sus talleres han tenido de una manera u otra un trabajo que le ha salido por ahí.* (Confeccionista 5, mujer, 34 años)

#### Prioridad en alimentación y salud, no en vestido

En la modificación de los hábitos de consumo de las personas, fruto de la crisis, la vestimenta no fue considerada como necesidad básica. Así, se redujeron las posibilidades de que los trabajadores ligados al sector textil se recuperaran rápidamente de las pérdidas generadas por la cuarentena.

*Durante la pandemia, los recursos o lo que realmente necesitábamos era la alimentación y salud, nada más. Entonces, ahora recién como que ya vamos dejando el tema de la pandemia y ya con los ingresos que tienen las familias les alcanza para comprarse algo de ropa; entonces, el negocio en Gamarra va incrementando y, por ende, hay trabajo para todos, bueno, para los que están direccionados a Gamarra, ¿no?; tanto confeccionistas, vendedores, cortadores, todo tipo.* (Confeccionista 2, varón, 38 años)

#### Trabajo sin jornada

Las personas que trabajaban en la confección que durante la cuarentena trasladaron sus talleres a sus hogares, percibieron cambios en su jornada laboral, de una jornada extendida pero fija a una jornada flexible, porque debían compartir horas de trabajo con las labores del hogar, así como procesaron el incremento de la carga laboral, ya que las personas que empleaban habían migrado de la ciudad a causa de la cuarentena.

*Acá en casa no tengo horario fijo, mayormente ya no salgo, estoy en casa trabajando y cuando me estreso un poco me voy con mi hijo; así es mi rutina, no tengo un horario fijo.* (Confeccionista 6, varón, 29 años)

*Gamarra abre sus puertas a las 08:00 a.m., pero la mayoría empieza a trabajar desde las 09:00 a.m. hasta las 19:00 p.m. Ese es el horario, pero ahora como estoy en casa, no me levanto a las 07:00 a.m. y trabajo hasta las 23:00 p.m. No tengo un horario de trabajo en casa, pero en Gamarra creo que cierra todas sus puertas principales a las 22:00 p.m. y hasta esa hora puedes estar ahí.* (Confeccionista 7, varón, 20 años)

#### Alimentación hogareña y sobrepeso

Con relación a los hábitos de alimentación, hubo un incremento en el consumo de alimentos que, sumado a la nula actividad física, ocasionó un incremento de peso. Por otro lado, se identificó un cambio en la valoración de la comida hogareña, porque la comida preparada en el hogar era considerada más saludable que la comida preparada en la calle.

*Físicamente engordé bastante, porque comíamos y dormíamos, no había actividad física. Solo por ratos había actividad física, porque de algún modo teníamos que distraernos, entonces hacíamos ejercicios, diferentes actividades, jugábamos y mirábamos vídeos*. (Confeccionista 4, varón, 24 años)

*Anteriormente en Gamarra podías comprar menú, pero ahora estoy en casa y convivo con mi mamá, y en casa se cocina más saludable, eso sí cambió mucho. En Gamarra tienes que comer lo que venden, así sea “chatarra” o lo que fuera.* (Confeccionista 7, varón, 20 años)

#### Cuidado e higiene

Las medidas sanitarias de cuidado familiar promovidas por el Estado, como el lavado de manos, el uso de alcohol, la limpieza frecuente de espacios y cambios de ropa, fueron percibidas como positivas e interiorizadas por las personas entrevistadas, convirtiéndose así en prácticas permanentes de higiene personal.

*Con el tema de higiene, cada vez que ibas a comprar al mercado se lavaba todas las verduras en una tina. Las lechugas se dejaban con un poco de lejía por varios minutos. Todo se desinfectaba con alcohol, desinfectaban en la puerta, los zapatos tenían que estar desinfectados, si tenías que salir por seguridad. Yo era el que más salía, uno por familia, como decía Vizcarra. Nos cambió la vida, cada vez que íbamos hacer algo, teníamos que hacer la limpieza del producto, después tenías que desinfectarlo, que todo esté limpio, que cada uno esté en su propio plato, que no se confunda. El cambio más radical fue el tema de limpieza ¿no?, que todo esté más pulcro.* (Confeccionista 14, varón, 24 años)

#### Más vida familiar

Los trabajadores entrevistados se caracterizaban por dedicar la mayor cantidad de horas de su vida al trabajo. Por ello, aquellos que antes de la pandemia tenían la costumbre de salir frecuentemente, o pasar tiempo en familia solo los fines de semana, fueron los más afectados por la cuarentena.

*Cambió bastante por el motivo que yo trabajaba en Gamarra, trabajaba de lunes a sábado, hacía deporte a veces, el domingo salía con familia al parque, pero hoy en día la libertad no está a un 100% como antes, antes había libertad de salir fin de semana, hasta viajar, pero hoy en día para viajar uno tiene un poco de miedo de contagiarse o de enfermarse, aparte también seguimos con la pandemia*. (Confeccionista 6, varón, 29 años)

Disponer de mayor tiempo en el hogar, por la cuarentena forzosa, posibilitó también la recuperación de espacios compartidos ligados al consumo de alimentos como el desayuno, el almuerzo y la cena, prácticas tradicionales familiares que habían sido abandonadas antes de la pandemia a causa del trabajo. En general, la pandemia fortaleció a la familia, porque la puso a prueba y promovió su adaptación a los nuevos retos^41^^,^^42^.

*Antes yo más paraba en Gamarra, prácticamente era como mi casa, porque ahí me quedaba, dormía ahí. Tenía que tender mi colchón ahí para dormir, porque no llegaba mucho a mi casa. En cambio, ahora es diferente. Mi hijo está en mi casa, está en su cuarto. En cambio, antes no, todo era en taller. Hoy en día voy al mercado y cocino en mi casa. Todo en mi casa. Creo que en esa parte avancé bastante, porque antes todo era en la calle. O sea, no compartía casi con familia mucho, porque todo era allá. En cambio, yo ahora cocino para todos. Tomamos desayuno en familia, almorzamos en familia, cenamos en familia y así.* (Confeccionista 9, mujer, 28 años)

#### Compra de provisiones

A medida que la cuarentena fue ampliándose, el gobierno estableció normas que regularon el horario de compra de víveres de consumo y de primera necesidad con la finalidad de evitar la aglomeración y la propagación del virus. Se probaron diversas estrategias como la compra cada 15 días, la compra una vez por semana, la restricción del desplazamiento por género, entre otras. Ello alteró la frecuencia de compra de las familias, más para varios días o incluso semanas, así como la variación del miembro responsable de realizar dicha tarea.

*Cuando el mercado estaba abierto hasta la 1 de la tarde, solo se podía salir en el transcurso de la mañana, entonces salía a comprar un rato una vez a la semana o una vez cada 15 días.* (Vendedor ambulante 5, varón, 63 años)

*Sola, nos turnábamos, un día iba un hermano, otra quincena iba otro y comprábamos para dos semanas los víveres, para no salir y contagiarnos.* (Vendedor ambulante 4, mujer, 45 años)

#### Menos interacción personal, más virtual

Durante la cuarentena se clausuró el funcionamiento de espacios de esparcimiento ocasionando que las personas no tuvieran espacios para interactuar de manera presencial con sus grupos de pares. Por esta razón, las personas entrevistadas más jóvenes y adultas migraron al mundo virtual y en estos espacios, incrementaron su cantidad de contactos. Si bien el contacto personal entre amigos se redujo, hubo relaciones de amistad que prevalecieron debido al uso de las redes sociales como medio de comunicación. Asimismo, otro grupo de personas entrevistadas utilizó estos espacios para la venta de sus productos, así como para el establecimiento de relaciones con sus clientes. La pandemia digitalizó más las relaciones sociales: la velocidad promedio de Internet de banda ancha fija en megabits por segundo (Mbps) saltó en Perú de 22,4 Mbps a 74,6 Mbps entre abril de 2018 y agosto de 2022^43^.

*Económicamente no estaba bien, entonces empecé a ver qué podía vender en ese momento, o la gente qué podría querer. Empecé a comprar poca cantidad de mamelucos, y mascarillas. Luego empecé a vender por Internet en “Marketplace” y poco a poco empecé a vender ropa también.* (Confeccionista 7, varón, 20 años)

*Ahora me contacto con mis amistades del trabajo y colegio por redes sociales, a través del celular nom*á*s. Casi como que no hay tiempo para ir a sus casas físicamente, solo nos contactamos por celular y por redes sociales.* (Confeccionista 10, mujer, 18 años)

*El cambio fue engancharse más a las redes sociales, porque antes de la pandemia la gente estaba enfocada en la tienda física/presencial, pero desde la pandemia hemos aprendido a acoplarnos a lo que son redes sociales. Ha sido una gran oportunidad, porque ahora sumo 50% de las redes sociales.* (Comerciante de tienda 15, mujer, 26 años)

#### Redistribución de las familias para el cuidado

La pandemia llevó a muchas familias a tomar decisiones sobre cómo redistribuirse entre los varios domicilios de los familiares, para cubrir mejor todas las necesidades de cuidado en la nueva situación. Si se tenían personas mayores en la familia, tendían a tener el mínimo contacto con ellos para prevenir posibles contagios, aun cuando eso significara no verlos buen tiempo. En otros casos, algunas personas entrevistadas tomaron la decisión de cambiar de residencia y vivir con sus familias de origen, o incluso volver a las provincias donde estaban sus mayores.

*El contacto con mi familia cambió. No verlos es o fue deprimente durante este tiempo; el año pasado no pude visitarlos, ni me pudieron visitar, me dio mucha pena. Ahora nos comunicamos por llamadas de vez en cuando.* (Vendedor ambulante 5, varón, 63 años)

*Con mi familia, se podría decir que ahora somos más apegados. Es decir, antes de la pandemia, dos o tres hermanos vivían en otra parte, otro convivía con su novia, pero cuando se declaró la pandemia tuvieron que venirse a la casa de mi mamá. Todos vivíamos en la misma casa.* (Confeccionista 7, varón, 20 años)

Una cantidad minoritaria de las personas entrevistadas vivía sola antes de la cuarentena y, a diferencia de quienes vivían en compañía, experimentaron sensaciones de aburrimiento, preocupación y estrés porque no podían salir ni tener contacto físico con amistades o familiares y su preocupación se incrementaba conforme se ampliaba la pandemia.

#### Vivir el covid-19 sin prueba diagnóstica

Entre las personas entrevistadas, el número de contagios por covid-19 con prueba diagnóstica fue bajo. No obstante, algunas de las personas entrevistadas tuvieron dificultades para determinar si los síntomas que experimentaron fueron ocasionados o no por el virus, ya que habían tenido contacto con personas que sí se contagiaron. La mayoría no tuvo una prueba diagnóstica. Esta incertidumbre sobre haber contraído o no el virus es un hallazgo similar al obtenido en otros estudios de corte cualitativo^44^.

*No me hice una prueba de descarte de covid-19, pero hace ocho meses, sentí una gripe, me dio fiebre, tuve pérdida del olfato y del gusto. Estuve dos días en mi cuarto encerrado, dos días que me dio fuerte, que me tumbo a la cama. Al tercer día había agarrado un poco de fuerza, estuve comiendo muchas bebidas calientes y sopa, eso me levantó, me sacó del virus*. (Vendedor ambulante 1, varón, 27 años)

*La verdad no sabría decir si era covid-19 o gripe, pero si estuve un poco de resfrió, dolor de estómago, todos los síntomas de los que dicen que tienen, pero no fue grave, fue un par de días, de ahí se me pasó*. (Confeccionista 8, mujer, 24 años)

#### La salud mental en el encierro

Con el pasar de los días, algunas personas afirmaron que comenzaron a sentir dolor de cabeza a causa de la ansiedad de no saber cuánto más se prolongaría el encierro, seguido de insomnio, tristeza, preocupación y depresión. Según las personas entrevistadas, la pandemia afectó la salud mental debido a los altos niveles de estrés, la preocupación constante e incertidumbre hacia el futuro y una de sus manifestaciones fue el cambio brusco de humor.

*Psicológicamente nos afectó bastante porque mucha gente moría y eso nos tenía preocupados, niños, jóvenes y adultos mayores morían. Además, la mayor concentración de caso covid-19 estaba en Lima, para nosotros que habíamos llegado de Cusco era algo preocupante*. (Confeccionista 4, varón, 24 años)

*Yo estuve diagnosticada de depresión antes de la pandemia. Sin embargo, la pandemia empeoró mi condición, me cayó muy fuerte, bien feo, porque quería salir y liberarme, pero no podía. Me la pasaba llorando en mi casa, no salía, hasta llegas a un punto de autolesionarte, porque no sabes qué hacer.* (Comerciante de tienda 1, mujer, 20 años)

## DISCUSIÓN

El estudio identifica cambios en la vida social en los tiempos de pandemia, referidos a la vivencia de una sucesión de shocks en cadena, la distorsión del tiempo y el espacio por el encierro, la crisis de las rutinas cotidianas previas, las nuevas estrategias de sobrevivencia, el recorte del consumo, el cambio en la vida laboral, el trabajo sin jornada, el trabajo extrahogareño en riesgo, la mayor presencia de la familia, la redistribución familiar para el cuidado, la priorización de la alimentación y salud, la alimentación hogareña, la no actividad física y sobrepeso, la preminencia de la higiene, la mayor interacción virtual, la incertidumbre de vivir el covid-19 sin prueba diagnóstica y la percepción de problemas de salud mental, entre otros.

La literatura sobre el tema ha identificado cambios similares y otros diferentes, en los diferentes ámbitos de la vida de las personas, las organizaciones y la sociedad en su conjunto^45^. Algunos han subrayado el recorte del consumo de alimentos y de la canasta familiar, así como cambios en los hábitos alimenticios^46^; la búsqueda de préstamos familiares o bancarios; el cambio de actividad económica; o el regreso a su lugar de origen^47^^,^^48^. Asimismo, diferentes estudios evidencian las nuevas dinámicas como el espacio compartido del trabajo y el cuidado en el hogar, sobre todo por parte de las mujeres^49^; el fortalecimiento de los vínculos familiares, que fueron antes relegados por el trabajo^50^.

En general, la literatura sobre la epidemia y pospandemia muestra que hay tres rutas sociales pospandémicas, no necesariamente alternativas: la primera, la continuación de las tendencias anteriores; la segunda, el surgimiento de desvíos limitados hacia patrones de conducta modificados; y la tercera, la instalación de nuevos hábitos sociales^51^. Lo específico de las sociedades informales, con poca presencia de un Estado protector es, conforme a nuestro estudio y otros, la velocidad y profundidad del giro de la vida social en estas situaciones críticas, pues se requieren mecanismos sustitutos de autosostenibilidad como condición de sobrevivencia. 

En esto, los enfoques generales sobre la dinámica y el cambio social son útiles para analizar lo sucedido. Kurt Levin^27^, psicólogo social, señala que los cambios sociales se producían en tres etapas: el “descongelamiento” de las pautas existentes, luego el movimiento hacia el cambio deseado y al final el “recongelamiento” de los nuevos patrones de conducta como institucionalidad. Berger y Luckman^28^ ilustraron cómo las sociedades son “construcciones sociales” y no realidades dadas e inamovibles. A la vez, se necesitan miradas más precisas, que articulen lo micro, meso y macro, para hilvanar los mecanismos, actores, contextos, tiempos y resultados del cambio, como las de la interacción social de Goffman o la centrada en el actor de Olivier de Sardan^29^. Este caso de la pandemia lo exige porque su particularidad fue la celeridad de la sustitución de unas pautas sociales por otras, debido a las cuarentenas obligatorias. Normalmente, la gente vive conforme a rutinas que automatizan la vida social y estas rutinas organizan las pautas sociales para cada ocasión. Hay, por tanto, un tiempo para que la socialización de los patrones de conducta social construya las personalidades sociales de los actores, como enseña Goffman, pero en este caso ese tiempo se aceleró. Además, la situación informal no era “descongelable” y era contradictoria con la inmovilización obligatoria. No toda la vieja normalidad podía, o debía, ser cambiada. En la interpretación goffmaniana hemos estado ante dos “escenificaciones” en paralelo, cada cual con sus rutinas, roles, estatus, funciones sociales, personajes, expresiones verbales y no verbales, relaciones sociales, conductas creyentes y cínicas, fachadas, modales, apariencias y auditorios. En la interpretación de Olivier de Sardan, estábamos ante una “revancha de los contextos”, en que la intervención originaba efectos no deseados^52^. Es lícito, por tanto, preguntarse en la pospandemia qué pautas podrían ser inerciales y cuales son reversibles, cuales convenientes e inconvenientes, y cuáles fueron una excepción que no se constituyeron en norma y cuáles sí. 

La pregunta actual es, por tanto: ¿la transformación de los hábitos cotidianos o de la sociabilidad de las personas con trabajos informales serán sostenibles en el tiempo pospandémico? ¿Qué es más perecible y qué podría ser más perdurable? La literatura optimista sobre la posibilidad de un giro en la sociabilidad del futuro acuñó el concepto de “nueva normalidad” para subrayar los cambios saludables propiciados por la pandemia que debieran permanecer^53^^,^^54^. La literatura pesimista ha planteado que esta nueva normalidad se parecerá mucho a la vieja normalidad^55^. Lo cierto es que hubo más optimismo al inicio de la pandemia que ahora, en la inicial pospandemia. La realidad mostró, como explicó Dávila, que aquellas personas con trabajos informales tuvieron que evaluar en plena crisis, si era mayor la amenaza del contagio o la de no tener ingresos^56^, por lo que, mientras algunas esperaron que pase el pico de la pandemia desde sus hogares, otras “no tuvieron otra opción que ponerse en peligro”^57^. Ello confirma la relevancia de definir, medir y trabajar el tema de vulnerabilidad económica que afectó (y afecta) a una proporción significativa de la población (34%, en 2019) y se estima en 14 puntos porcentuales por encima de la población en pobreza^58^.

Al final, la reapertura de todas las actividades se hizo, como era previsible, restaurando la “vieja normalidad”, por una sociedad exhausta que no tenía otro camino que el retorno a las prácticas sociales previas, antes que marchar a una vida social todavía en formación y que nunca aparece de conjunto sino en forma incremental. Sin embargo, dado que es probable que pasemos a un virus endémico, con picos en estaciones del año y zonas, a nuevas pandemias y policrisis, posiblemente el futuro tendrá elementos adquiridos de la vida cotidiana con el covid-19, hacia una sociedad más preventiva ante los riesgos emergentes. Estudios sobre las configuraciones sociales de los próximos años nos iluminarán sobre si los elementos positivos que se vivieron se han convertido en tendencias firmes o si se han ido diluyendo, en particular los anuncios de conductas más higienistas y más consideradas con la salud, más cercanas a la familia, con un uso mayor de la digitalización y trabajo híbrido, con más capital social, esto es, si tendremos una sociabilidad más adaptable ante la adversidad o si no habremos aprendido la lección.
